# Endocrine Aspects of 4H Leukodystrophy: A Case Report and Review of the Literature

**DOI:** 10.1155/2015/314594

**Published:** 2015-05-31

**Authors:** Emma Billington, Geneviève Bernard, William Gibson, Bernard Corenblum

**Affiliations:** ^1^Division of Endocrinology & Metabolism, University of Calgary, 1820 Richmond Road SW, Calgary, AB, Canada T2T 5C7; ^2^Bone & Joint Research Group, Department of Medicine, University of Auckland, Private Bag Box 92019, Auckland 1020, New Zealand; ^3^Departments of Pediatrics, Neurology and Neurosurgery, Division of Pediatric Neurology, Research Institute of the McGill University Health Centre, 1001 boul Décarie, Site Glen Pavilion E / Block E, Montréal, QC, Canada H4A 3J1; ^4^Department of Medical Genetics, University of British Columbia, Child and Family Research Institute, 950 West 28th Avenue, Vancouver, BC, Canada V5Z 4H4

## Abstract

*Introduction*. 4H leukodystrophy is an autosomal recessive RNA polymerase III-related leukodystrophy, characterized by hypomyelination, with or without hypodontia (or other dental abnormalities) and hypogonadotropic hypogonadism. *Case Presentation*. We describe a 28-year-old female who presented with primary amenorrhea at the age of 19. She had a history of very mild neurological and dental abnormalities. She was found to have hypogonadotropic hypogonadism, and magnetic resonance imaging of the brain showed hypomyelination. The diagnosis of 4H leukodystrophy was made. She was subsequently found to have mutations in the *POLR3B* gene, which encodes the second largest subunit of RNA polymerase III. She wished to become pregnant and failed to respond to pulsatile GnRH but achieved normal follicular growth and ovulation with subcutaneous gonadotropin therapy. *Discussion*. Patients with 4H leukodystrophy may initially present with hypogonadotropic hypogonadism, particularly if neurological and dental manifestations are subtle. Making the diagnosis has important implications for prognosis and management. Progressive neurologic deterioration is expected, and progressive endocrine dysfunction may occur. Patients with 4H leukodystrophy should be counseled about disease progression and about this disease's autosomal recessive inheritance pattern. In those who wish to conceive, ovulation induction may be achieved with subcutaneous gonadotropin therapy, but pulsatile GnRH does not appear to be effective.

## 1. Introduction

4H leukodystrophy is one of five overlapping leukodystrophies that have been associated with mutations in the* POLR3A* and* POLR3B* genes, which encode the two largest subunits of RNA polymerase III (Pol III) [1–6]. The 4H leukodystrophy is characterized by hypomyelination, with or without hypodontia (or other dental abnormalities), and hypogonadotropic hypogonadism [7, 8]. Patients typically present in childhood with neurological dysfunction and/or dental abnormalities. The hypogonadotropic hypogonadism typically manifests in adolescence when patients fail to demonstrate normal pubertal development [7–13].

Previous discussion of the endocrine aspects of 4H leukodystrophy has been limited, and induction of ovulation in patients with 4H leukodystrophy has not been described yet. Here, we report on a female patient who presented for endocrine evaluation with primary amenorrhea and was found to have 4H leukodystrophy. We review the clinical, biochemical, and genetic features of 4H leukodystrophy. In addition, we discuss management of the endocrine manifestations of 4H leukodystrophy, in light of our experience with ovulation induction in this patient.

## 2. Case Presentation

**Figure 1:**
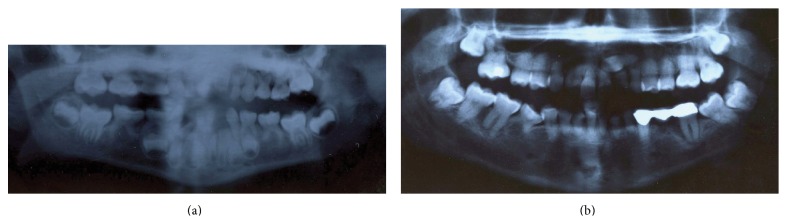
Dental X-rays performed at the age of 6 (a) and the age of 19 (b). The lower second bicuspids are absent. There are two supernumerary teeth underneath her secondary lower incisors, which may represent ectopic, malformed bicuspids.

A 19-year-old female presented to our clinic with primary amenorrhea. Thelarche and pubarche had occurred at the age of 13. She denied any symptoms suggestive of primary ovarian insufficiency, polycystic ovarian syndrome, pituitary pathology, or functional hypothalamic disease. Her history was significant for mild dysarthria since childhood. She had been diagnosed with congenital absence of the lower second bicuspids, and dental X-rays (see Figure 1) had identified two supernumerary teeth underneath her secondary lower incisors, which may have represented ectopic, malformed bicuspids. She was myopic in both eyes, requiring eyeglasses. She was otherwise healthy and had exhibited normal development as a child. She had completed high school and denied having significant academic difficulties. There was no family history of delayed puberty, other forms of endocrine dysfunction, consanguinity, or inherited disorders.

On examination, her height was 154.3 cm (10th percentile) and her BMI was 27.0 kg/m^2^. She had no dysmorphic features. She had Tanner stage 5 breast and pubic hair development and no features of pituitary hormone deficiency. Neurological examination revealed gaze-evoked nystagmus, a mildly ataxic gait, and mild spasticity of the upper limbs. She had no dysmetria or dysarthria. She did not have a tremor.

**Figure 2:**
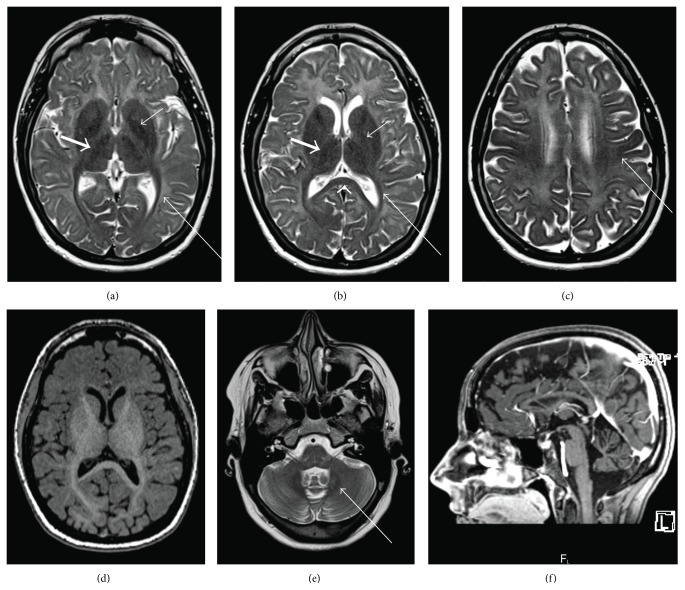
MRI of the brain performed at the age of 28, demonstrating evidence of a hypomyelinating leukodystrophy. Compared to grey matter structures, there is diffuse hyperintensity of the white matter on T2 weighted images (a, b, c), with hyperintense signal on T1 weighted images (d), consistent with hypomyelination. As previously reported, there is characteristic relative preservation of myelination within the following structures: optic radiations (long thin white arrow; a, b), anterolateral nucleus of the thalamus (thick white arrow; a, b), globus pallidus (short thin white arrow; a, b), posterior corpus callosum (white arrow head; b), corticospinal tracts (white arrow; c), and the dentate nucleus (white arrow; e). Sagittal T1 weighted imaging (f) demonstrates thinning of the corpus callosum and mild cerebellar atrophy.

Laboratory investigations included LH < 1 (reference range (RR) 1–13 IU/L), FSH 5 (RR 2–10 IU/L), estradiol 62 (prepubertal range: 0–130 pmol/L), prolactin 25 (0–25 *μ*g/L), and free T4 14.9 (8.0–22.0 pmol/L). Her karyotype was 46, XX. She did not menstruate after a progesterone challenge but did when given the oral contraceptive pill, indicating that she was hypoestrogenized and that she did not have an outflow abnormality. She had no further breast growth while being on the oral contraceptive pill. Pelvic ultrasound showed a small uterus, measuring 4.5 × 3.0 × 1.4 cm, normal endometrium, and small ovaries, measuring 1.5 × 0.9 × 1.1 cm (0.78 cm^3^) on the left and 1.7 × 2.4 × 1.2 cm (2.56 cm^3^) on the right (average normal volume 6-7 cm^3^) [14]. MRI of the sella revealed mildly decreased pituitary bulk. A full MRI of the brain was not obtained at the time of initial evaluation, but a subsequent MRI (see Figure 2) revealed diffuse hypomyelination (i.e., hyperintense white matter on T2 weighted images compared to grey matter structures) [15, 16] as well as a slightly thin corpus callosum and mild atrophy of the cerebellum, predominantly seen at the vermis. 4H leukodystrophy was suspected based on the patient's clinical and radiological features. Subsequent DNA sequencing revealed that she is a compound heterozygote for* POLR3B* mutations, specifically c.1568T>A (amino acid change p.V523E) and the rare intronic variant c.2817+30T>A [5].

At the age of 24, the patient desired pregnancy and so the oral contraceptive pill was discontinued. Trials of both subcutaneous and intravenous pulsatile GnRH therapies failed to show biochemical evidence of follicular development, with a peak estradiol level of 56 pmol/L. She went on to achieve normal follicular development in response to gonadotropin therapy, with a peak estradiol level of 1581 pmol/L and development of a dominant follicle measuring 1.86 cm. She did not conceive. She decided not to undergo further ovulation induction therapy, and so hormone replacement therapy was reinitiated at physiologic doses.

The patient was reassessed at the age of 27. At that time, she had no clinical evidence of progressive neurological dysfunction or pituitary hormone deficiency. A morning cortisol level was 860 mmol/L, free T4 was 14.3 pmol/L, and serum IGF-1 level was 207 (117–329 *μ*g/L). Given that she did not have any symptoms suggestive of growth hormone or adrenocorticotropic hormone deficiency, dynamic testing was not undertaken. At the age of 28, a detailed, gadolinium enhanced MRI of her head was obtained (Figure 2). Appearance was consistent with her diagnosis of 4H leukodystrophy, demonstrating diffuse hyperintensity of the white matter compared to grey matter structures on T2 weighted images and hyperintensity of the deep white matter on T1 weighted images. Several areas demonstrated relative preservation of myelination, as described in Pol III-related or 4H leukodystrophy [7, 16, 17].

## 3. Discussion

Our case demonstrates that patients with 4H leukodystrophy may present for endocrine evaluation due to hypogonadotropic hypogonadism before the diagnosis of Pol III-related leukodystrophy is made. Recognizing the syndrome and making the diagnosis of 4H leukodystrophy are crucial, as they have important genetic, neurological, and reproductive implications. Here, we review the salient features of 4H leukodystrophy with a focus on the endocrine aspects.

### 3.1. Etiology

4H leukodystrophy is a genetic disorder that displays an autosomal recessive inheritance pattern [8]. Mutations in* POLR3A* and* POLR3B*, which encode the two largest subunits of RNA polymerase III (Pol III), have now been identified in several patients with 4H leukodystrophy [5–7, 18]. Pol III transcripts consist of small untranslated RNA molecules that are involved in crucial cellular functions such as transcription and translation [19]. Several of these transcripts have been found to have brain-specific expression, which may account for the profound central nervous system effects with relative sparing of other organs that is seen in Pol III-related leukodystrophies [20]. It has been proposed that mutations in* POLR3A* and* POLR3B* result in hypomyelination by altering the levels of the small RNAs required for the normal development of white matter [18].

In addition to a mutation in exon 15 of the* POLR3B* gene, which is relatively common amongst patients with 4H leukodystrophy caused by* POLR3B* mutations, our patient also has a unique intronic mutation at intron 24 [5], which has not been described in others with 4H leukodystrophy. The effect of this intronic variation on the encoded protein has not been fully elucidated, although it most likely leads to a frameshift due to creation of a cryptic acceptor splice site [5].

### 3.2. Clinical and Biochemical Manifestations

The presentation of 4H leukodystrophy is variable, but most patients present in early childhood with motor delay or regression and are often subsequently found to have dental manifestations [7]. The majority of patients also exhibit pronounced myopia [7]. The dental abnormalities seen in 4H leukodystrophy may be overt, with several teeth failing to develop, or they may be very subtle, requiring X-rays for identification [10, 11, 21]. This patient had congenital absence of both mandibular second bicuspids but had also been found to have two supernumerary teeth underneath her secondary mandibular incisors; it is uncertain whether these truly represent supernumerary teeth or whether they are ectopic, malformed bicuspids.

Cerebellar dysfunction is the most frequent neurological abnormality [7, 10–13, 21], and the majority of patients also have cognitive dysfunction [1, 6, 7, 9, 12, 18, 22]. Progressive neurological deterioration is a characteristic feature of patients with 4H leukodystrophy and other Pol III-related leukodystrophies, with many patients becoming wheelchair-bound and exhibiting significant cognitive impairment by the time they reach young adulthood [1, 7, 10, 13, 18, 21, 23]. However, the age-of-onset of this neurological dysfunction is variable. Whether the severity of early developmental delays (when present) correlates with the onset and rapidity of later neurological decline is not yet known.

Patients with 4H leukodystrophy have delayed puberty, low baseline LH and FSH levels, and no response to pituitary stimulation with GnRH [11–13]. The majority of females with 4H leukodystrophy lack spontaneous pubertal development, suggesting that hypogonadotropic hypogonadism is usually established prior to adolescence in this disorder [12, 13]. Although hypogonadism has not been recognized until early adulthood in some male patients, normal initial pubertal development followed by progressive hypogonadism has not been described [9, 10]. Some patients also have growth hormone deficiency, which was shown to be progressive in one individual [9, 12]. The other pituitary hormones do not appear to be affected.

Our patient's presentation is unusual in comparison to reports of other patients with 4H leukodystrophy. Her dental and neurological manifestations were exceptionally subtle and did not lead to diagnosis prior to puberty. She does not have evidence of significant cognitive dysfunction and has not exhibited neurological deterioration during eight years of follow-up. In addition, although she failed to go through menarche, she did have spontaneous thelarche at the age of 13. This indicates that she was estrogenized at that time, strongly suggesting that her gonadotropin insufficiency developed after the age of 13. The relatively mild clinical course of our patient may be explained in part by her intronic variant, which may produce some normal* POLR3B* protein if the cryptic splice site is not used.

### 3.3. Diagnosis

Diagnosis is made based on a combination of clinical features and characteristic findings on dental X-rays and on magnetic resonance imaging (MRI) of the brain [7, 15, 16]. A diagnosis can be strongly suspected without mutation analysis, but genetic sequencing should be utilized now that causative genes have been identified.

We have demonstrated that neurological and dental manifestations may be minimal, and patients with 4H leukodystrophy may present for endocrine evaluation due to hypogonadotropic hypogonadism without a prior diagnosis. By looking for clinical manifestations of hypomyelination and hypodontia in patients who present with hypogonadal hypogonadism without a clear etiology, endocrinologists may be able to facilitate a diagnosis of 4H leukodystrophy. Making this diagnosis has important implications in terms of prognosis, genetic counseling, and management from an endocrine, dental, neurological, and ophthalmological perspective.

### 3.4. Management of Endocrine Dysfunction

Given the broad clinical manifestations of 4H leukodystrophy, most patients will require coordinated care from several subspecialists, including a dentist, neurologist, clinical geneticist, and endocrinologist. The endocrine aspects of management for this disorder include surveillance for progressive endocrine dysfunction, consideration and initiation of hormone replacement therapy, and induction of ovulation or spermatogenesis if the patient desires fertility. Given the rarity of this disorder, the optimal method of surveillance for progressive endocrine dysfunction is unclear. Screening for clinical evidence of ACTH, GH, and TSH deficiency at least every few years may be prudent, as may undertaking biochemical evaluation of pituitary hormone function when suggestive clinical features are present. In terms of correcting reproductive hormone deficiencies, we suggest that the same principles of hormone replacement therapy used in patients with other forms of hypogonadotropic hypogonadism can be applied to patients with 4H leukodystrophy. Orcesi et al. describe a 12-year-old boy with 4H leukodystrophy who did not respond to a GnRH stimulation test. They then treated him with chorionic gonadotropin and noted that his height velocity increased substantially and his testosterone levels normalized [11]. Our female patient tolerated physiologic ovarian steroid replacement and had withdrawal bleeds as expected.

Fertility treatment in patients with 4H leukodystrophy brings up some considerations that are unique to this disorder, but there is little evidence to guide management. At the time that ovulation induction therapy was initially offered to our patient, neither the inheritance pattern nor the neurological implications of 4H leukodystrophy were well understood. Therefore, she did not receive genetic counseling nor was her partner offered carrier testing. However, with the current state of knowledge of this disorder, we recommend that genetic counseling be offered. Patients should be aware of the propensity for neurologic deterioration in 4H leukodystrophy and the autosomal recessive inheritance pattern. Given the rarity of this disorder, carrier testing in partners has not been described but could be considered as sequencing for the causative mutations becomes more readily available.

In terms of choice of therapy, our patient underwent ovulation induction therapy with both pulsatile GnRH and gonadotropin therapy. She did not exhibit a rise in serum estradiol levels or evidence of follicular development on transvaginal ultrasound while receiving a total of three cycles of pulsatile GnRH therapy, a therapy to which more than 90% of women with hypothalamic amenorrhea will respond [24]. She was subsequently switched to subcutaneous gonadotropin therapy and showed biochemical and radiographic evidence of normal follicular development. These results imply that the defect causing the hypogonadotropic hypogonadism seen in 4H leukodystrophy is at the level of the pituitary, potentially due to abnormal small RNA synthesis, resulting in defective transcription of key mediators required for the function of the GnRH receptor protein, or for gonadotropin synthesis. Therefore, it appears that gonadotropins should be first line therapy for ovulation induction in women with this syndrome.

### 3.5. Conclusions

4H leukodystrophy should be considered in the differential diagnosis of patients who present with hypogonadotropic hypogonadism and no overt cause, particularly if dental or neurological abnormalities are present. Endocrine dysfunction in these patients includes hypogonadotropic hypogonadism and GH deficiency and may be progressive, requiring ongoing surveillance. Progressive neurological deterioration is also a hallmark. The defect causing hypogonadism in 4H leukodystrophy appears to be at the hypophyseal level and precludes the use of pulsatile GnRH for ovulation induction, whereas subcutaneous gonadotropin therapy appears to be effective. Reports of 4H leukodystrophy are rare, and the diagnosis may be missed if clinical manifestations are subtle. However, making the diagnosis is critical for ensuring that appropriate counseling, therapy, and surveillance are initiated.
